# FEASIBILITY OF AEROBIC FITNESS TESTING IN AMNESTIC MILD COGNITIVE IMPAIRMENT FROM THE ACT TRIAL

**DOI:** 10.1093/geroni/igad104.3713

**Published:** 2023-12-21

**Authors:** Fang Yu, Dereck Salisbury, Feng Lin

**Affiliations:** Arizona State University, Phoenix, Arizona, United States; University of Minnesota, Minneapolis, Minnesota, United States; Stanford University, Stanford, California, United States

## Abstract

Aerobic fitness has been postulated as a physiological mechanism of action for aerobic exercise to modify Alzheimer’s disease, but it has not been adequately studied in older adults with (MCI). The purpose of this study is to evaluate the feasibility of a gold-standard, laboratory-based, symptom-limited cycle-ergometer exercise testing (GXT) in older adults with amnestic mild cognitive impairment (aMCI) to assess peak oxygen consumption (VO2max [ml/kg/min]) using baseline data from the ACT Trial. The test was categorized as a submax, near-max, and max test based on if 0, 1, and ≥2 of the American College of Sports Medicine criteria was met: >90% of age-predicted maximum heart rate, plateau in VO2Peak, respiratory exchange ratio >1.10, and rating of perceived exertion >17. VO2max was predicted as 3.5*maximal metabolic equivalents (METs). Among 146 enrolled participants (73.8±5.7 years old with 16.9±2.9 years of education, 51.4% men, and 91.8% Caucasian), 145 completed GXTs. Sixteen GXTs (11.0%) were submax (VO2Peak 13.5±4.4), 53 (36.6%) were near-max (VO2Peak 16.5±4.1), and 76 (52.4%) were max (VO2Peak 18.4±5.1). Predicted VO2max were 21.6±6.8, 28.7±26.3, and 30.0±20.5 from submax, near-max, and max tests, respectively. Among max GXTs, VO2max was 19.5±4.9 in men (n=37) and 17.4±5.1 in women (n=39). In conclusion, GXTs was feasible for ~52% of the participants. METs-based prediction overly inflates VO2max estimates, in comparison to VO2max obtained from max GXTs. VO2max levels are substantially lower in older adults with aMCI than peers (23.1±6.3 in men and 21.1±3.4 in women), suggesting aerobic exercise as an important intervention for this population.

